# A Plant Proteinase Inhibitor from *Enterolobium contortisiliquum* Attenuates Pulmonary Mechanics, Inflammation and Remodeling Induced by Elastase in Mice

**DOI:** 10.3390/ijms18020403

**Published:** 2017-02-14

**Authors:** Osmar Aparecido Theodoro-Júnior, Renato Fraga Righetti, Rafael Almeida-Reis, Bruno Tadeu Martins-Oliveira, Leandro Vilela Oliva, Carla Máximo Prado, Beatriz Mangueira Saraiva-Romanholo, Edna Aparecida Leick, Nathalia Montouro Pinheiro, Yara Aparecida Lobo, Mílton de Arruda Martins, Maria Luiza Vilela Oliva, Iolanda de Fátima Lopes Calvo Tibério

**Affiliations:** 1 Department of Clinical Medicine, School of Medicine, University of São Paulo, 01246-903 São Paulo, Brazil; osmartheodorojr@gmail.com (O.A.T.-J.); refragar@gmail.com (R.F.R.); dosreisrafael@gmail.com (R.A.-R.); brunotad@gmail.com (B.T.M.-O.); leandrooliva@hotmail.com (L.V.O.); beatrizmsaraiva@gmail.com (B.M.S.-R.); leick51@yahoo.com.br (E.A.L.); pinheiro.nathalia@gmail.com (N.M.P.); mmartins@usp.br (M.d.A.M.); 2 Physical Therapy Department, Hospital Sírio-Libanês, 01308-050 São Paulo, Brazil; 3 Department of Bioscience, Federal University of Sao Paulo, 09972-270 Diadema, Brazil; cmaximoprado@gmail.com; 4 Department of Biochemistry, Federal University of São Paulo, 09972-270 Diadema, Brazil; yara.lobo@yahoo.com.br (Y.A.L.); olivamlv@gmail.com (M.L.V.O.)

**Keywords:** COPD, emphysema, proteinase inhibitor, EcTI

## Abstract

Proteinase inhibitors have been associated with anti-inflammatory and antioxidant activities and may represent a potential therapeutic treatment for emphysema. Our aim was to evaluate the effects of a plant Kunitz proteinase inhibitor, *Enterolobium contortisiliquum* trypsin inhibitor (EcTI), on several aspects of experimental elastase-induced pulmonary inflammation in mice. C57/Bl6 mice were intratracheally administered elastase (ELA) or saline (SAL) and were treated intraperitoneally with EcTI (ELA-EcTI, SAL-EcTI) on days 1, 14 and 21. On day 28, pulmonary mechanics, exhaled nitric oxide (ENO) and number leucocytes in the bronchoalveolar lavage fluid (BALF) were evaluated. Subsequently, lung immunohistochemical staining was submitted to morphometry. EcTI treatment reduced responses of the mechanical respiratory system, number of cells in the BALF, and reduced tumor necrosis factor-α (TNF-α), matrix metalloproteinase-9 (MMP-9), matrix metalloproteinase-12 (MMP-12), tissue inhibitor of matrix metalloproteinase (TIMP-1), endothelial nitric oxide synthase (eNOS) and inducible nitric oxide synthase (iNOS)-positive cells and volume proportion of isoprostane, collagen and elastic fibers in the airways and alveolar walls compared with the ELA group. EcTI treatment reduced elastase induced pulmonary inflammation, remodeling, oxidative stress and mechanical alterations, suggesting that this inhibitor may be a potential therapeutic tool for chronic obstructive pulmonary disease (COPD) management.

## 1. Introduction

Chronic obstructive pulmonary disease (COPD) is the major pulmonary cause of chronic morbidity and is one of the most important causes of death in most countries [1]. The physiopathology of this disease, characterized by a mixture of small airway disease (obstructive bronchiolitis) and parenchymal destruction (emphysema), leads to obstructive processes and airflow limitations [2]. Pulmonary emphysema is defined as enlargement and destruction of lung parenchyma, including respiratory bronchioles, alveolar ducts and alveoli [3]. According to the proteinase-antiproteinase hypothesis, cigarette smoke causes inflammation and the subsequent release of proteolytic enzymes into the lung in excess of their natural inhibitors. In this way, elastase secreted by neutrophils and macrophages plays an important role in alveolar wall destruction [3,4].

The approach to the management of COPD is divided in terms of the reduction of risk factors and the therapeutic management of stable disease and of exacerbations [5]. No currently available treatments reduce the progression or even adequately suppress the inflammation in the small airways and lung parenchyma. To better understand the pathogenesis of emphysema, the elastase-induced model was developed 30 years ago and is a simple and widely accepted method to induce emphysema in animals. Since then, this model has been used to elucidate the mechanisms involved in emphysema pathophysiology and to test new therapeutic agents [6].

One of the new approaches to emphysema treatment that has been discussed is the use of proteinase inhibitors, which may inhibit proteolytic enzymes or increase endogenous antiproteinases contributing to the prevention of the progression of the disease [7]. Proteinases are important signaling molecules that are involved in physiological processes such as hemostasis, cell death, cell proliferation, DNA replication, inflammatory response and tissue remodeling [8]. Some proteinase inhibitors are found in plants and their biological and pathological function, in the prevention of unwanted proteolysis, have been studied extensively [9,10].

*Enterolobium contortisiliquum* (Leguminosae-Mimosoideae) is tree widely cultivated in many tropical countries. From its seeds, several proteins were isolated and structurally and biochemically characterized as EcTI, the *Enterolobium contortisiliquum* trypsin inhibitor, a polyspecific inhibitor belonging to plant Kunitz inhibitor family [11,12].

The present study aimed to evaluate if the plant Kunitz proteinase inhibitor, EcTI contributes to the inactivation of elastase-induced mechanical, inflammatory, and oxidative stress and remodeling alterations in an experimental, elastase-induced pulmonary inflammation mouse model. In addition, alterations of both the alveolar walls and airway are part of this disease model and the effects on these structures may differ depending on treatment. To elucidate the effects of EcTI treatment in these different compartments in an experimental model of emphysema, we evaluated the extent of inflammation, remodeling and oxidative stress in the alveolar walls and airways using immunohistochemistry.

## 2. Results

### 2.1. Lung Mechanics

**Figure 1:**
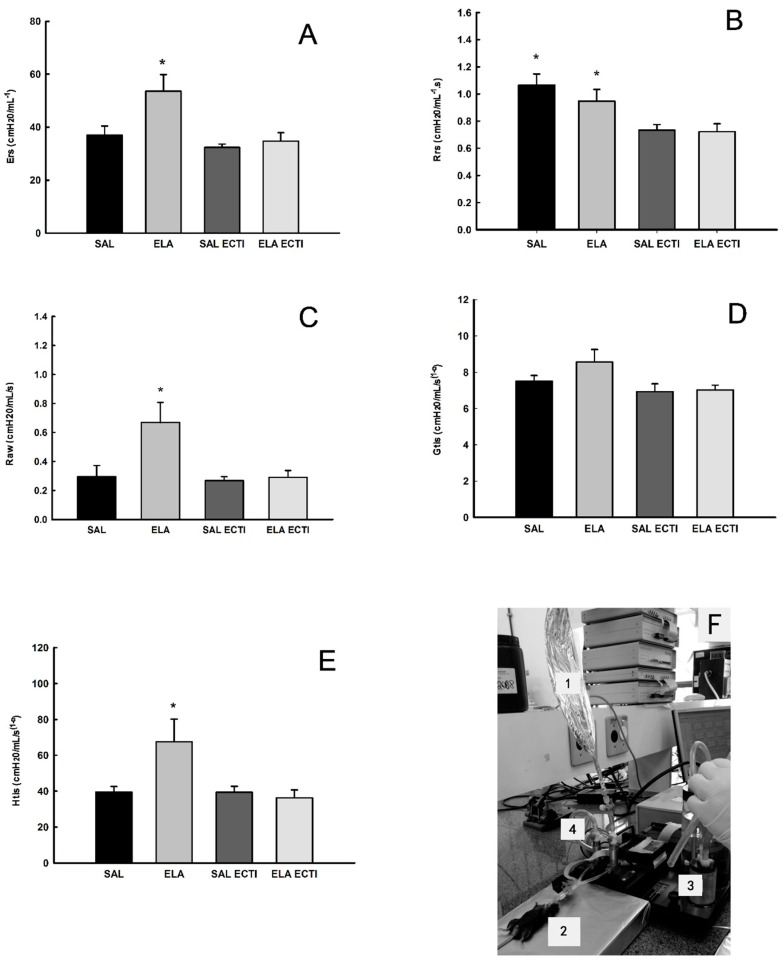
(**A**) Median and standard error (SE) of respiratory system elastance (*E*_rs_) for all experimental groups. * *p* < 0.05, compared with the saline (SAL), SAL-*Enterolobium contortisiliquum* trypsin inhibitor (EcTI) and elastase (ELA)-EcTI groups; (**B**) Median and SE of respiratory system resistance (*R*_rs_) for all experimental groups. * *p* < 0.05, compared with the SAL-EcTI and ELA-EcTI groups; (**C**) Median and standard error of *R*_aw_ for all experimental groups. * *p* < 0.05, compared with the SAL, SAL-EcTI and ELA-EcTI groups; (**D**) Mean and SE of *G*_tis_ for all experimental groups. There were no significant differences among the groups; (**E**) Median and SE of *H*_tis_ for all experimental groups. * *p* < 0.05, compared with the SAL, SAL-EcTI and ELA-EcTI groups; (**F**) Photograph of the ENO and the mechanical evaluation: (1) Mylar bag for exhaled nitric oxide (ENO); (2) animal connected to a mechanical ventilator; (3) mechanical ventilator for small animals (Flexivent) and (4) nitric oxide filter.

Figure 1A shows the values of respiratory system elastance (*E*_rs_) in all experimental groups. There was a significant increase in *E*_rs_ in the elastase (ELA) groups when compared to the saline (SAL) control and SAL-EcTI groups (*p* < 0.05). EcTI tratment reduced the *E*_rs_ in the ELA-EcTI group compared with the ELA group (*p* < 0.05).

Figure 1B shows the values of respiratory system resistance (*R*_rs_) in all experimental groups. There was a significant decrease in *R*_rs_ in the ELA-EcTI and SAL-EcTI groups when compared with the ELA and SAL groups (*p* < 0.05). There were no differences between the ELA and SAL groups as well as between SAL-EcTI and ELA-EcTI groups.

Figure 1C shows the values of airway resistance (*R*_aw_) in all experimental groups. There was a significant increase in *R*_aw_ in the ELA group when compared with the SAL and SAL-EcTI groups (*p* < 0.05). EcTI treatment reduced *R*_aw_ in the ELA-EcTI group compared with the ELA group (*p* < 0.05). There were no differences between the ELA-EcTI and SAL-EcTI groups.

Figure 1D shows the values of lung tissue damping (*G*_tis_) in all experimental groups. There was no difference among the groups.

Figure 1E shows the values of lung tissue elastance (*H*_tis_) in all experimental groups. There was a significant increase of *H*_tis_ in the ELA group when compared with the SAL and SAL-EcTI groups (*p* < 0.05). EcTI treatment reduced the *H*_tis_ in the ELA-EcTI group compared with the ELA group (*p* < 0.05). There were no differences between the ELA-EcTI, SAL and SAL-EcTI groups.

### 2.2. Bronchoalveolar Lavage Fluid (BALF)

The total and differential inflammatory cell counts are shown in Table 1. The ELA group presented an increase in total inflammatory cells, macrophages, neutrophils, lymphocytes and eosinophils, compared with the SAL group (*p* < 0.05). Both cell counts were significantly reduced in the ELA-EcTI and SAL-EcTI groups compared with the ELA group (*p* < 0.05).

### 2.3. Morphometric Analysis

#### 2.3.1. Mean Linear Intercept (Lm)

**Figure 2:**
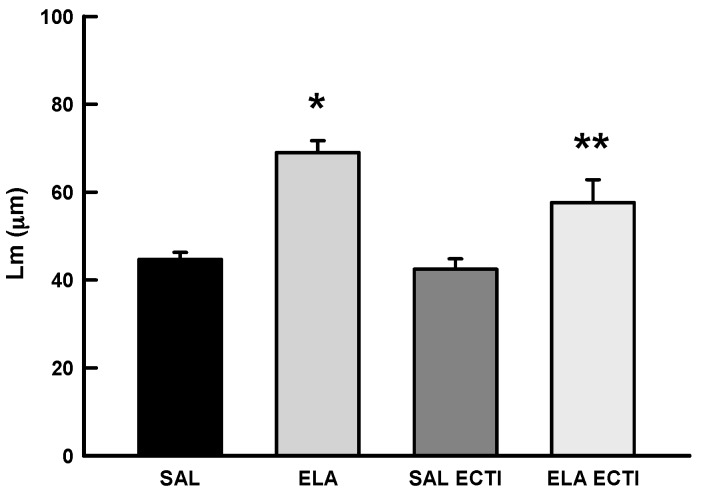
Mean and SE of Lm values for the four experimental groups. * *p* < 0.05, compared with the SAL and SAL-EcTI groups. ** *p* < 0.05, compared with the ELA group.

Figure 2 shows the values of the Lm in all experimental groups. There was a significant increase in the Lm in the ELA group compared with the SAL and SAL-EcTI groups (*p* < 0.05). EcTI treatment reduced the Lm in the ELA-EcTI group compared with the ELA group (*p* < 0.05). There were no differences between the SAL and SAL-EcTI groups.

#### 2.3.2. Lung Inflammation

The absolute values of inflammatory markers for alveolar walls and airways in all experiment groups are shown in Table 2 and Table 3, respectively.

There was an increase in neutrophils, macrophages and tumor necrosis factor-α (TNF-α) positive cells in the alveolar walls of mice in the ELA group compared with the control groups (*p* < 0.05). EcTI treatment reduced the number of neutrophils, macrophages and TNF-α-positive cells in the alveolar walls in animals that received elastase (ELA-EcTI group) compared with the ELA group (*p* < 0.05). There were no differences between the SAL, SAL-EcTI and ELA-EcTI groups.

We obtained similar results in the airways of mice. There was an increase in the number of neutrophils and TNF-α-positive cells in the airways of mice in the ELA group compared with the SAL and SAL-EcTI groups (*p* < 0.05). EcTI treatment in the ELA-EcTI group reduced the number of neutrophils and TNF-α positive cells in the airway compared with the ELA group (*p* < 0.05). The SAL-EcTI group was different when compared to the SAL and ELA-EcTI groups (*p* < 0.05).

#### 2.3.3. Extracellular Matrix Remodeling

The absolute values of remodeling markers for alveolar walls and airways in all experimental groups are shown in Table 2 and Table 3, respectively.

There was an increase in the volume proportion of collagen fibers in the alveolar walls of mice in the ELA group compared with the SAL and SAL-EcTI groups (*p* < 0.05). In the ELA-EcTI group, there was a reduction in the volume proportion of collagen and elastic fibers compared with the ELA group (*p* < 0.05). There were no differences between the SAL, SAL-EcTI and ELA-EcTI groups. There was also an increase in the volume proportion of elastic fibers in the ELA group compared with the SAL and SAL-EcTI groups (*p* < 0.05). In the ELA-EcTI group, there was a reduction in the volume proportion of elastic fibers compared with the ELA group, but there were no statistically significant differences. There were no differences between the SAL, SAL-EcTI and ELA-EcTI groups. Similarly, when evaluating airway remodeling the volume proportion of collagen and elastic fibers in the ELA group increase compared with the SAL and SAL-EcTI groups (*p* < 0.05). EcTI treatment (ELA-EcTI group) reduced the volume proportion of collagen and elastic fibers compared with the ELA group (*p* < 0.05).

In evaluating matrix metalloproteinase (MMP)-9, MMP-12 and tissue inhibitor matrix metalloproteinase-1 (TIMP-1) positive cells in the alveolar walls, we observed an increase in MMP-9, MMP-12 and TIMP-1 positive cells in the ELA group compared with the SAL, SAL-EcTI and ELA-EcTI groups (*p* < 0.05). EcTI treatment (ELA-EcTI group) reduced the number of MMP-9, MMP-12 and TIMP-1 positive cells compared with the ELA group (*p* < 0.05). The SAL-EcTI group was different when compared to the SAL and ELA-EcTI groups (*p* < 0.05). We obtained similar results when we evaluated MMP-9, MMP-12 and TIMP-1-positive cells in airways. There was an increase in MMP-9 and MMP-12-positive cells in the ELA group compared with the SAL and SAL-EcTI groups (*p* < 0.05). EcTI treatment (ELA-EcTI group) reduced the number of MMP-9 and MMP-12-positive cells compared with the ELA group (*p* < 0.05). The SAL-EcTI group was different when compared to the SAL and ELA-EcTI groups (*p* < 0.05). However, in the airways, there was an increase in TIMP-1-positive cells in the ELA and ELA-EcTI groups compared with the SAL and SAL-EcTI groups (*p* < 0.05). There were no differences between the ELA and ELA-EcTI groups.

#### 2.3.4. Oxidative Stress

The absolute values of oxidative stress markers for alveolar walls and airways in all experimental groups are shown in Table 2 and Table 3, respectively. There were an increases in inducible nitric oxide synthase (iNOS) and endothelial nitric oxide synthase (eNOS)-positive cells in the alveolar walls of mice in the ELA group compared with the SAL and SAL-EcTI groups (*p* < 0.05). In the ELA-EcTI group we observed a reduction in the number of iNOS and eNOS-positive cells compared with the ELA group (*p* < 0.05). There were no differences between the SAL, SAL-EcTI and ELA-EcTI groups.

We observed similar results in the airways of the mice analysed; there was an increase in iNOS and eNOS-positive cells in the ELA group compared with the SAL and SAL-EcTI groups (*p* < 0.05). In the ELA-EcTI group, there was a reduced number of iNOS and eNOS positive cells compared with the ELA group (*p* < 0.05). There were no differences between the SAL, SAL-EcTI and ELA-EcTI groups.

The evaluation of the volume proportion of 8-isoprostane-prostaglandin 2α (8-*iso*-PGF2α) in alveolar walls and airways showed that there was an increased volume proportion of 8-*iso*-PGF2α in the ELA group compared with the SAL and SAL-EcTI groups (*p* < 0.05). In the ELA-EcTI group, we noticed a reduction in the volume proportion of 8-*iso*-PGF2α compared with the ELA group (*p* < 0.001). There were statistically significant differences between the ELA-EcTI group compared with the SAL and SAL-EcTI groups (*p* < 0.05).

**Figure 3:**
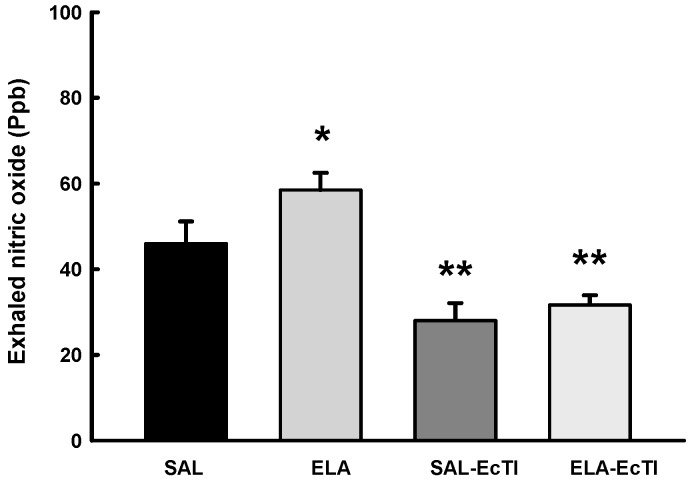
Mean and standard error of exhaled nitric oxide (ENO) for the four experimental groups. * *p* < 0.05, compared with the SAL and SAL-EcTI groups. ** *p* < 0.05, compared with the SAL and ELA groups.

Figure 3 shows the values of exhaled nitric oxide (ENO) in all experimental groups. There was a significant increase of ENO in the ELA group compared with the SAL group (*p* < 0.05). EcTI treatment reduced the ENO values in the ELA-EcTI and SAL-EcTI groups (*p* < 0.05). There was a decrease in ENO in the SAL-ECTI group when compared with the SAL group (*p* < 0.05).

#### 2.3.5. Tracheobronchial Positive Mucin Cells

**Figure 4:**
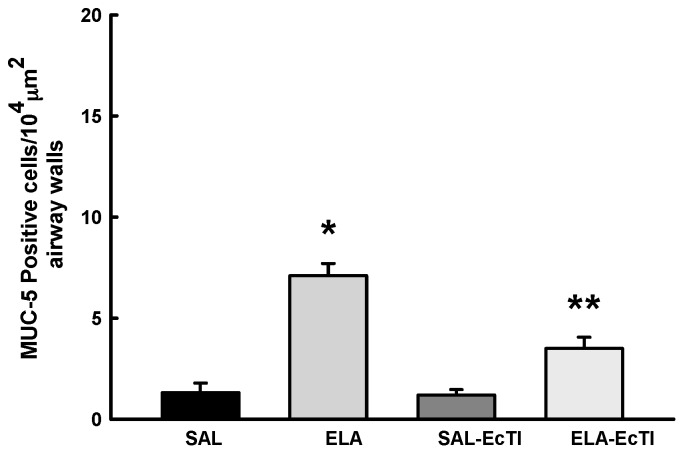
Mean and SE of positive MUC-5AC cells in the airways of animals in all experimental groups. * *p* < 0.05, compared with the SAL and SAL-EcTI groups. ** *p* < 0.05, compared with the ELA group.

The number of positive MUC-5AC cells was evaluated only in the airways and is shown in Figure 4.

There was an increase in MUC-5AC positive cells in the airways in the ELA group compared with the SAL and SAL-EcTI groups (*p* < 0.05). In the ELA-EcTI group we observed a reduction in the number of MUC-5AC-positive cells compared with the ELA group (*p* < 0.001). There were statistically significant differences between the ELA-EcTI group compared with the SAL and SAL-EcTI groups (*p* < 0.05).

### 2.4. Qualitative Analysis

**Figure 5:**
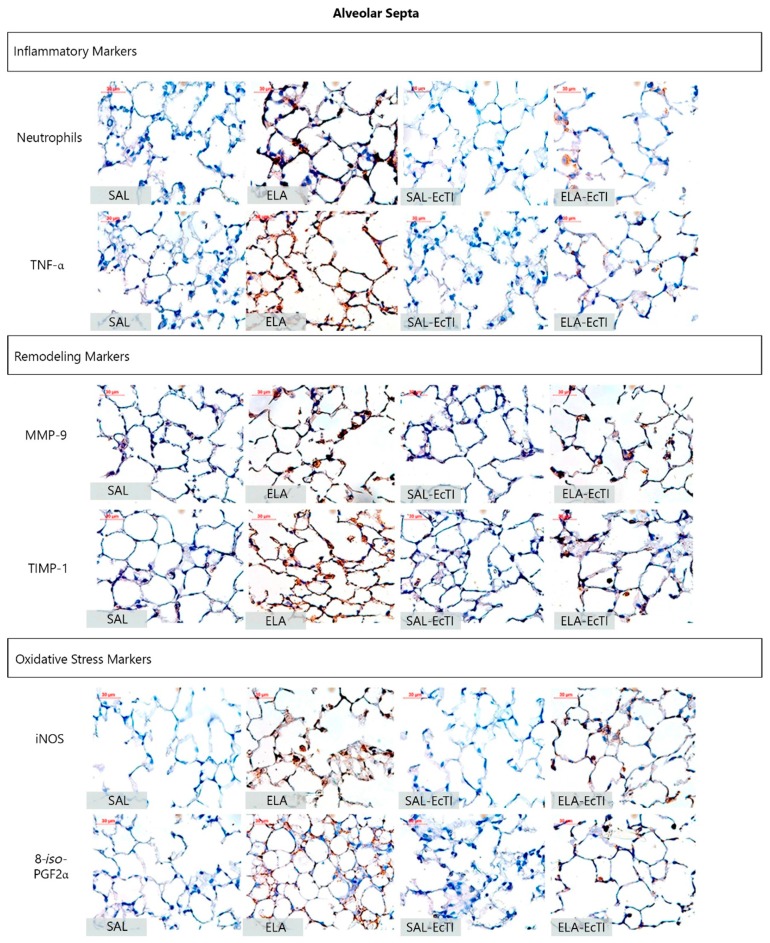
Inflammatory, remodeling and oxidative stress markers in the alveolar walls: photomicrographs of the results of the immunohistochemical analyses of the extracellular matrix inflammatory, remodeling and oxidative stress process in the alveolar walls to detect neutrophils, TNF-α, MMP-9, TIMP-1, iNOS and 8-*iso*-PGF2α. Magnification of 400×. All the following experimental groups are represented: SAL, ELA, SAL-EcTI and ELA-EcTI. We observed that the proteinase inhibitor, EcTI attenuated the cellular response induced by elastase. Scale bar = 30 µm.

**Figure 6:**
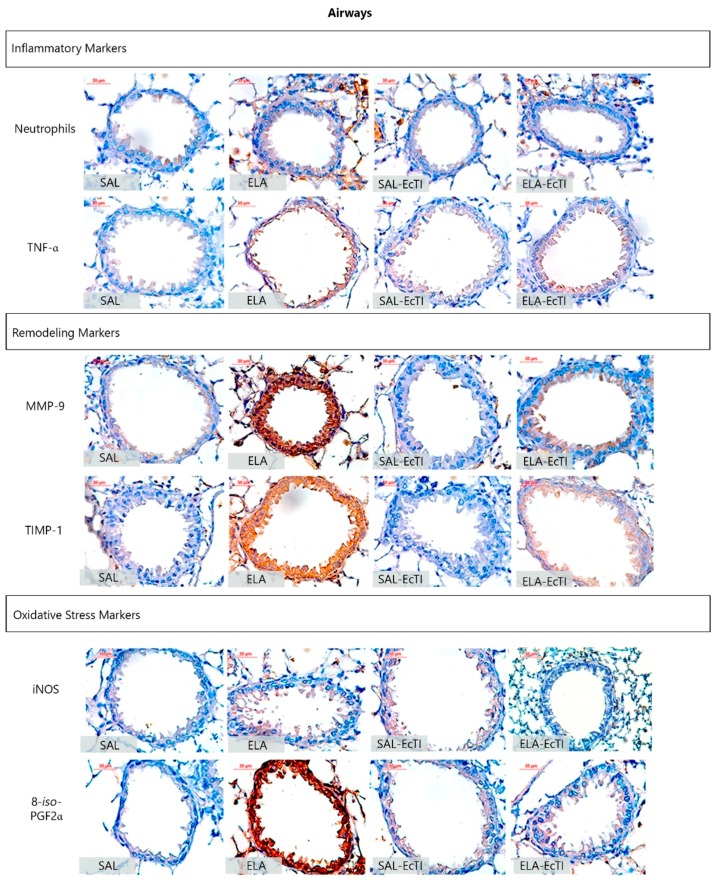
Inflammatory, remodeling and oxidative stress markers in the airway walls; photomicrographs of the results of the immunohistochemical analyses of the inflammatory, remodeling and oxidative stress process in the airway walls used to detect neutrophils, TNF-α, MMP-9, TIMP-1, iNOS and 8-*iso*-PGF2α. Magnification of 400×. All the following experimental groups are represented: SAL, ELA, SAL-EcTI and ELA-EcTI. We observed that the proteinase inhibitor EcTI attenuated the cellular response induced by elastase. Scale bar = 30 µm.

Figure 5 and Figure 6 show representative photomicrographs of the inflammatory, remodeling and oxidative stress processes as reflected by neutrophils and other cells stained positive for TNF-α, MMP-9, TIMP-1, iNOS and 8-*iso*-PGF2α in the alveolar walls and airways, respectively.

The ELA group showed an intense infiltration of neutrophils and cells positive for TNF-α, MMP-9, TIMP-1, iNOS and volume fraction of 8-*iso*-PGF2α that suggested lung inflammation, remodeling and oxidative stress processes. Both treatments this number of positive cells in the alveolar walls and airway, as observed in representative photomicrographs.

## 3. Discussion

In the present study, we tested the theory that the proteinase inhibitor from *Enterolobium contortisiliquum* (EcTI) would attenuate elastase-induced pulmonary alterations in mice. We showed that EcTI decreased functional parameters evaluated in lung mechanics, inflammation, extracellular matrix remodeling and the oxidative stress response in the alveolar septum and airways walls in this model of lung injury induced by intratracheally administered elastase.

According to the proteinase-antiproteinase hypothesis, new therapeutic strategies aiming to control the production and/or inactivation of proteases are being investigated for the treatment of chronic obstructive pulmonary diseases [13]. Proteinases are no longer considered just enzymes for protein degradation; instead, they are now considered important signaling molecules involved in vital biological processes and proteinase inhibitors are being intensively studied [8]. Synthetic molecules, such as CP-471, 474, ZD0892, SP-B, MR889 and FR901277, have been developed to test the therapeutic role of proteinase inhibitors in COPD [14,15,16,17,18].

The relevance of the effect of proteinases in emphysema is widely accepted. However, the specific cells and/or proteinases that have key functions in the development and progression of COPD remain a matter of discussion and controversy. Some authors suggest that the serine, cysteine and metalloproteinase classes of proteinases are the classes most likely to be involved in the pathogenesis of COPD [19,20,21]. Neutrophil elastase, proteinase 3 and cathepsin G are serine class proteinases, stored by polymorphonuclear cells (PMN) and monocytes, and released when pro-inflammatory mediators induce PMN degranulation. These proteinases are often related with lung parenchyma destruction and mucus production. The cysteine class is represented by cathepsin S and L which are potent elastases, and help macrophage-mediated extracellular matrix degradation. The metalloproteinase class is represented by MMP1, 2, 9 and 14 and disintegrin and metalloprotease (ADAM). In addition to promoting collagen and elastin degradation, metalloproteinases also increase MUC-5AC epithelial expression [22,23].

We investigated the functional modulation of EcTI treatment in this mouse model of lung injury induced by intratracheally administered elastase by analyzing lung mechanics. Our results showed that EcTI treatment reduced the respiratory system resistance (*R*_rs_) and the elastance (*E*_rs_). EcTI also reduced the tissue resistance elastance (*H*_tis_) and the airway resistance (*R*_aw_). These results suggest that intratracheally administered elastase changed the functionality of both the airways and the lung parenchyma.

The reduction in the respiratory system resistance (*R*_rs_) was observed in both the SAL-EcTI and ELA-EcTI groups. These results suggest that EcTI might have an airway bronchodilator effect that should be tested in future studies using experimental models of asthma. This bronchodilator effect is confirmed by the airway resistance (*R*_aw_) data, where EcTI reduced airway resistance in animals receiving either elastase or saline.

Respiratory system elastance (*E*_rs_) increased in animals that received elastase. These data are corroborated by the pulmonary *H*_tis_ values that maintained the same pattern of response. The *R*_aw_, *E*_rs_ and *H*_tis_ values demonstrated an effect of EcTI treatment on both the distal airways and alveolar septa.

Different lung mechanic effects have been observed in elastase animal models. Hantos et al. [24], measuring the pulmonary mechanics in mice induced by via intratracheal elastase administration showed an increase in volume and a decrease in lung tissue elastance, but no changes in airway and tissue resistance. Thus, they concluded, unlike others, that the destruction of the lung tissue is not associated with pulmonary dysfunction.

Ito et al. [25] administered porcine pancreatic elastase by nebulization to evaluate the lung mechanics of mice pulmonary emphysema. They showed a decrease in respiratory system elastance and suggested that the abnormal remodeling of collagen plays a major role in pulmonary function and in the mechanical forces associated with emphysema. However, Scuri et al. [26] showed that elastase leads to increased production of bradykinin that increases respiratory system resistance and elastance and that the bradykinin B2 receptor antagonist blocked this response. These data suggest that the kallikrein-kinin system is involved. It is important to note that, although these studies used elastase to induce lung injury, the administration protocols and doses were different.

We noticed that the experimental model used was effective in increasing the alveolar spaces, as reflected by the Lm results. EcTI attenuated the destruction caused by intratracheal elastase-induced lung injury since the ELA-EcTI group had results similar to the controls.

Shapiro et al. [21] showed that animals that did not produce elastase tended to have an approximately 59% reduction in tissue destruction (as measured by Lm) compared with animals without this genetic alteration. The study exposed genetically modified mice that did not produce neutrophil elastase to cigarette smoke. Suzuki et al. [27], studying the effects of curcumin in an animal model of emphysema, showed that the yellow pigment from turmeric reduced the Lm in both elastase-induced lung injury and chronic exposure to cigarette smoke. Guarnieri et al. [16], using a high dose of elastase in mice showed that a peptide-elastase inhibitor which way bound to trifluoromethyl prevented expansion of the alveoli that had received elastase.

Evaluating bronchoalveolar lavage fluid is one of the most common and efficient methodologies for monitoring pulmonary inflammation [28]. The number of inflammatory cells in the BALF from mice in the ELA group increased in our study probably because of the role of neutrophil elastase on the displacement of leukocytes to the site of inflammation. We found significant differences in the numbers of all cells in the BALF from animals in the ELA and ELA-ECTI groups. There was a significant reduction in the number of total cells, macrophages, neutrophils, lymphocytes and eosinophils in those mice that received elastase and EcTI compared with those that did not, which indicates an important anti-inflammatory action of the protein inhibitor, similar to that reported by Neuhof et al. [29]. The Kunitz plant elastase inhibitor decreased swelling to the same degree as a reference substance used for the treatment of COPD. The authors concluded that this type of protein inhibitor could be a useful tool to study the role of neutrophil elastase in pathophysiological processes.

Testing the effect of curcumin in a model of elastase-induced emphysema, Suzuki et al. [27] observed that curcumin, which is a member of the ginger family, significantly reduced the number of macrophages, neutrophils and the total number of cells in the bronchoalveolar lavage fluid of mice that had received intratracheal elastase.

Plantier et al. [30] reported a similar results to Suzuki et al. [27]. Plantier et al. [30] evaluated the influence of keratinocyte growth factor (KGF) on mice that had received intratracheal elastase and showed that KGF significantly decreased the total number of cells, the number of macrophages and neutrophils in the BALF.

The number of neutrophils, macrophages and TNF-α positive cells were also evaluated in alveolar septa to assess the inflammatory response. We observed that EcTI was able to decrease the number of macrophages, neutrophils and TNF-α positive cells in alveolar septa. EcTI also reduced the number of neutrophils and TNF-α positive cells in airway walls.

To assess changes in extracellular matrix remodeling, we measured the volume fraction of collagen and elastic fibers, and the number of cells that were positive for, MMP-9, MMP-12 and TIMP-1 in alveolar septa and airway walls. EcTI treatment reduced the volume fraction of elastic and collagen fibers in the alveolar septa and airway walls.

Comparing two models of elastase or smoke induced lung injury in mice, Lopes et al. [31] showed that elastase-induced mice had higher values in elastin than animals exposed to 6 months of smoke. Collagen type I had expression the same pattern but the authors did not find significant differences between the groups. Nevertheless, mice exposed to smoke for 6 months had higher collagen type III expression than elastase-induced mice. EcTI treatment decreased the number of positive MMP-9 and MMP-12 cells in both the alveolar septa and airway walls. Hanaoka et al. [32] studied the effects of carbocisteine in exposed to cigarette smoke extracts and observed an increase in MMP-9 expression in lung tissue that was reduced with carbocisteine treatment. Shapiro et al. [21] using MMP-12 knock-out mice, showed that MMP-12 has an important role in the development of cigarette smoke-induced emphysema. Although high levels of MMP-9 increase TIMP-1 levels to restore homeostasis, we did not observe any effect of EcTI on TIMP-1 levels.

Since mucus hypersecretion is an important component in COPD pathophysiology and the proteinases play a key role in this process, we evaluated the number of MUC-5AC positive cells in airway walls and found that EcTI reduced the number of MUC-5AC positive cells. Liu et al. [33] evaluated the role of human airway trypsin-like protease (HAT) and protease-activated receptor 2 (PAR2) in MUC-5AC expression and found that both HAT and PAR2 stimulate MUC-5AC expression. They suggest that PAR2 could be a novel therapeutic target to control mucus hypersecretion.

In the present study, we demonstrated that intratracheal elastase administration caused an increase in exhaled nitric oxide (ENO), which is associated with an increased in the number of macrophages, neutrophils, lymphocytes and eosinophils. The treatment with the proteinase inhibitor EcTI attenuated this response, which was also associated with a reduction in the recruitment of inflammatory molecules and cells.

EcTI decreased the number of iNOS and eNOS-positive cells, a measure of the oxidative stress response, in both the alveolar septa and airway walls. EcTI treatment also reduced the volume fraction of 8-*iso*-PGF2α in the alveolar septa and airway walls. Prado et al. [34] observed that iNOS inhibition attenuated the hyperresponsiveness in animals with chronic allergic inflammation. Together these results may explain the change in airway resistance that we observed since iNOS plays a key role in the modulation of airway tone. These effects appear to be related to the effect of iNOS on Rho-kinase activity.

The present study had some limitations. An analysis of the toxicity of EcTI was not performed; nevertheless, several experimental models have used this inhibitor and no adverse effects of EcTI were identified [11,35]. Further studies are necessary to assess toxicity. Our results support the importance of EcTI treatment in the mechanics, lung inflammatory response, extracellular matrix remodeling, and oxidative stress in the lungs. Additional studies are needed to elucidate the mechanisms responsible for these changes.

## 4. Materials and Methods

### 4.1. Plant Protein

Inhibitor purification was assessed by reversed-phase chromatography in a C18 column and by 5%–12% sodium dodecyl sulfate (SDS)-polyacrylamide gel electrophoresis. The single band of the inhibitor obtained by the purification process used was similar to that described by de Paula et al., [11]. The concentration of the inhibitor was determined by the Lowry assay, and the dose used (2 mg/kg) was the same as that of the elastase inhibitor BbCI, another proteinase inhibitor [29].

### 4.2. Animals

Thirty-two young adult male C57Bl6 mice (20–25 g), obtained from the University of São Paulo were used. The animals were 6–8 weeks old and held through a period setting in the laboratory vivarium for 2 weeks prior to the study. The animals were handled in accordance with the guidelines for Care of Laboratory Animals (National Institutes of Health, publication 86–23, revised 1985). The study was approved by the Ethics Committee for Analysis of Research Projects (CAPPesq) of the Clinical Hospital and Faculty of Medicine—University of São Paulo (number: 243/10, 11 March 2010).

### 4.3. Animal Model

**Figure 7:**
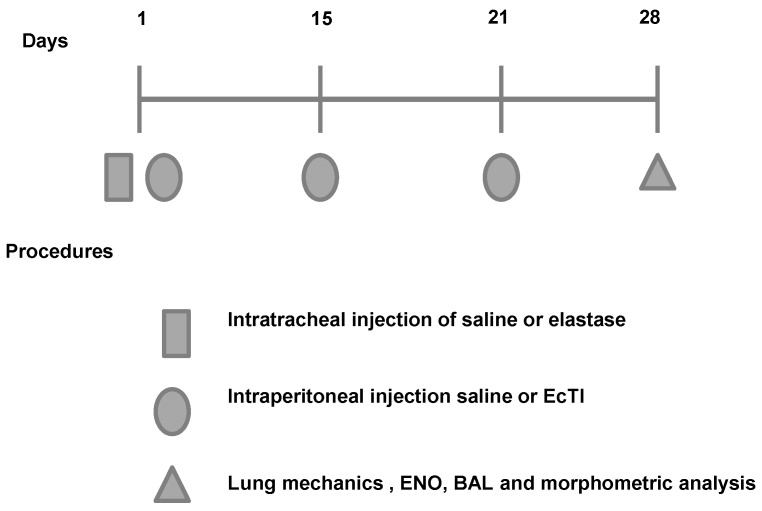
Timeline of the experimental protocol. On the first day of the protocol, animals received intratracheal instillation of elastase or vehicle. 2 h after the intratracheal instillation, animals received an intraperitoneal injection of the treatment (EcTI or saline). On the fifteenth day, animals received the second dose of treatment (EcTI or saline). On the twenty-first day, animals received the last dose of saline or EcTI.

Timeline of the experimental protocol are shown in Figure 7. The animals were randomly divided into 4 groups:

(1) ELA group: the animals were anesthetized with isoflurane, and the neck region was shaved and disinfected with Povidone. An incision was made approximately 0.5 cm in the mid-region of the neck to expose the trachea. Using a 30-unit syringe, a single 50 µL dose of 0.025 mg of porcine pancreatic elastase (E-1250-100 mg elastase solution pancreatic porcine pancreas type 2x crystallized aqueous solution contains approximately 0.01% thymol 14.29 mL, 7 mg of protein/M 5.2 units/mg protein; Sigma, Carlsbad, CA, USA) was intratracheally administered between the cartilaginous rings. Afterward, the cervical incision was closed with a 5.0 silk suture and disinfected with Povidone [34];
(2) ELA-EcTI group: 1 h after the intratracheal administration of elastase the mice received intraperitoneally injection (i.p.) of EcTI (2 mg/kg). On days 15, 21 and 28, the animals received two doses of EcTI (2 mg/kg i.p.) (*n* = 8);
(3) SAL group: 1 h after the intratracheal administration of saline (50 µL). On days 15, 21 and 28, the animals received two doses of saline i.p. (*n* = 8);
(4) SAL-EcTI: 1 h after the intratracheal administration of saline (50 µL), the mice received intraperitoneally injection of EcTI (2 mg/kg). On days 15, 21 and 28, the animals received two doses of EcTI (2 mg/kg i.p.) (*n* = 8).

### 4.4. Measurement of Exhaled Nitric Oxide (ENO) and Mechanical Evaluation

On day 28 of the experimental protocol, the animals were anesthetized with thiopental (250 mg/kg i.p), tracheotomized and ventilated at 150 breaths/min with a tidal volume of 10 mL/kg, and a positive end expiratory pressure (PEEP) of 5 cm H_2_O was applied using a mechanical ventilator (FlexiVent, Scireq, Montreal, QC, Canada). Next, ENO concentration was measured with a collection Mylar bag was attached to the expiratory output of the ventilator for 10 min and attached to the inspiratory breathing circuit input an NO filter. Afterward, using chemiluminescence fast-responding analyzer (NOA 280; Sievers Instruments Inc., Boulder, CO, USA) calibrated with an NO (nitric oxide) source certified 47-parts per billion (ppb) (White Martins, São Paulo, Brazil) and a zero NO filter (Sievers Instruments Inc.).

For mechanical evaluation of the respiratory system resistance (*R*_rs_) and elastance (*E*_rs_) values were obtained using the equation of motion of the respiratory system:

(1) $$Ptr(t)=ErsV(t)+RrsV^{\prime }(t)$$

We obtained the piston volume displacement (*V*_cyl_) was corrected to obtain the actual volume in the animals (*V*), cylinder pressure (*P*_cyl_) was corrected based on the value of Pao (open airway pressure) and flow (*V*′) by deriving V with respect to time. Next, animals muscle paralysis was induced by i.p. injection of pancuronium bromide (0.2 mg/kg) and experimental data from the forced oscillation technique were obtained. The ventilator produced flow oscillations for 16 s at different frequencies (from 0.25 to 19.625 Hz), using the constant phase model and a pop-up signal of 75% in 16 s. We obtained airway resistance (*R*_aw_), tissue damping (*G*_tis_) and tissue elastance (*H*_tis_) parameters and 3 blocks of 8 s were used to calculate the parameters of the mechanical oscillation [36], according to the following equation:

(2) $$Z(f)=R_{aw}+i{\left(2\pi f\right)}_{law}+\left[G_{tis}-iH_{tis}\right]/{\left(2\pi f\right)}^{\alpha }$$

In this experimental model, *Z*(*f*): impedance of air as a function of frequency, *i*: imaginary unit (−1½), *f*: frequency, law: inertance of the airways and

(3) $$\alpha =\left(2/\pi \right)\times \arctan\left(H_{tis}/G_{tis}\right)$$

### 4.5. Bronchoalveolar Lavage Fluid

BALF was performed by injecting a total of 1.5 mL of saline (3 × 0.5 mL) through a tracheal cannula. The total number of cells obtained in the lavage was determined using a Neubauer counting chamber. The differentiation of cells was performed using cytospin preparation followed by staining with Quick-Stain reagent. A differential cell count was performed by evaluating more than 300 cells with an optical microscope [36].

### 4.6. Lung Histology

Afterward mechanical respiratory system evaluated, lungs were removed en bloc and the lungs at a constant pressure of 20 cmH_2_O for 48 h to homogenize the distension of the pulmonary parenchyma were fixed with 4% formaldehyde for 24 h and after that were stored in 10% formalin for up to 7 days for morphometric studies and histological analysis. The slides were stained with Hematoxylin-Eosin, Resorcin-Fuchsin (method for elastic fibers identification) and Picrosirius Red (method for collagen fibers identification).

Using a Leica DM2500 microscope (Leica Microsystems, Wetzlar, Germany), a digital camera (Leica DFC420 Leica Microsystems, Wetzlar, Germany) and the image analysis software Image Proplus 4.5 (NIH, Silver Spring, MD, USA), we evaluated the collagen and elastic fiber content in the alveolar walls and airways. Five airways and fifteen alveolar walls fields at 400× magnification were evaluated for each animal. Collagen and elastic fiber content were evaluated in the interest area with a predetermined filter. The positive area of collagen and elastic fibers was expressed as a percentage of the total area of the airway wall or alveolar septum in the interest field. The slides were coded, and the researcher who performed the measurements was blinded [37].

### 4.7. Immunohistochemistry

Slices were deparaffinized and rehydrated for immunohistochemistry and were treated with Proteinase K for 20 min (37 °C), followed by 20 min at room temperature and washed in phosphate-buffered saline (PBS). For blocking endogenous peroxidases was performed by incubation with 3% hydrogen peroxide (H_2_O_2_) 10V (3 × 10 min) and slides sections of experimental and control tissue (positive and negative) were incubated overnight with primary antibodies.

Immunohistochemistry was performed with the following anti-bodies:: anti-mouse macrophage Mac2 (Cedarlane Lab, Burlington, ON, Canada; 1:60,000), anti-mouse neutrophils (AbD Serotec, Kidlington, UK; 1:400), anti-mouse MMP-9 (Santa Cruz Biotechnology, Dallas, TX, USA; 1:500), antibody anti-mouse MMP-12 (Santa Cruz Biotechnology, 1:100), anti-iNOS (LabVision, NeoMarkers, Fremont, CA, USA; 1:500), anti-eNOS (LabVision, NeoMarkers; 1:100), anti-TIMP-1 (LabVision, NeoMarkers; 1:400), anti-TNF (Santa Cruz Biotechnology; 1:300,), anti-isoprostane-8 (Oxford Biomedical Research, Oxford, UK; 1:10,000), and anti-MUC5AC (LabVision, NeoMarkers; 1:400).

Next, the slides were washed in PBS and incubated with secondary antibody using an ABCKit by Vectastain (Vector Elite-PK-6105 (anti-goat) or PK-6101 (anti-rabbit) (Vector Laboratories, Burlingame, CA, USA). For visualization of the positive cells the slides were washed in PBS and proteins were visualized using 3,3′-diaminobenzidine chromogen (DAB) (Sigma Chemical Co., St. Louis, MO, USA). The slides sections were counterstained with Harris hematoxylin (Merck, Darmstadt, Germany) and mounted using Entellan microscopy resin (Merck). Cells that stained positive as neutrophils or for MMP-9, MMP-12, TIMP-1, iNOS, eNOS, TNF-α, 8-*iso*-PGF2α and MUC-5AC were evaluated by the point-counting technique [38]. The positive cells in the alveolar walls and airways were with the conventional morphometric analysis using a reticule of known area (50 lines and 100 points) coupled in a microscope (CH30, Olympus, Tokyo, Japan). As the reticule should be placed in 10 random fields in the distal lung and 3–5 airway wall per animal at a magnification of 1000× and the number of positive cells was determined as the number of positive cells in each field divided by the number contacting the alveolar wall or airway area (10^4^ μm^2^). The researcher who performed the measurements was unaware of the study groups [38,39,40].

### 4.8. Mean Alveolar Diameter (Lm)

The alveolar diameter analysis was performed using a reticule of known area (50 lines and 100 points) coupled in a microscope (CH30). As the reticule should be placed in over the area of the lung parenchyma, and the intersections of the points in the alveolar wall were counted, excluding vessels and airways. Thus, the mean alveolar diameter was calculated according to the ratio of the number contacting the alveolar walls are to the number of intersections between the lines and the alveolar walls [36]. The researcher who performed the measurements was unaware of the study groups

### 4.9. Data Analyses

Statistical analysis was performed using Sigmastat software (SPSS Inc., Chicago, IL, USA). Multiple comparisons were made by one-way ANOVA. For comparisons between groups, we used the Holm–Sidak test. Data are presented as the mean ± standard error and are depicted in bar graphs with error bars. Data were considered significant when *p* < 0.05.

## 5. Conclusions

We have demonstrated that EcTI treatment attenuated the mechanics, inflammatory response, extracellular matrix remodeling and oxidative stress responses in the lungs in this experimental model of lung injury induced by the intratracheal administration of elastase. EcTI proved to be a promising therapeutic strategy for the treatment of emphysema, although further studies are needed to elucidate the mechanisms involved.

## Figures and Tables

**Table 1:** Absolute values of the number of cells in bronchoalveolar lavage fluid (BALF).

Inflammatory Markers	SAL	ELA	SAL-EcTI	ELA-EcTI
Total cells (10^4^ cells/mL)	0.51 ± 0.05	1.83 ± 0.06 *	0.61 ± 0.07	0.92 ± 0.3 **
Macrophages (10^4^ cells/mL)	0.46 ± 0.11	1.57 ± 0.17 *	0.57 ± 0.09	0.64 ± 0.12 **
Neutrophils (10^4^ cells/mL)	0.00 ± 0.00	0.05 ± 0.17 *	0.01 ± 0.09	0.00 ± 0.00 **
Lymphocytes (10^4^ cells/mL)	0.00 ± 0.00	0.36 ± 0.01 *	0.00 ± 0.00	0.00 ± 0.00 **
Eosinophils (10^4^ cells/mL)	0.00 ± 0.00	0.15 ± 0.01 *	0.00 ± 0.00	0.00 ± 0.00 **

The total cells, macrophages, neutrophils, lymphocytes and eosinophils are expressed in 10^4^ cells/mL. * *p* < 0.05, compared with the SAL and SAL-EcTI groups; ** *p* < 0.05, compared with the ELA group. SAL, saline; ELA, elastase; EcTI, *Enterolobium contortisiliquum* trypsin inhibitor.

**Table 2:** Absolute values of the morphometric analysis for inflammatory, remodeling and oxidative stress markers in the alveolar walls.

**Inflammatory Markers**	**SAL**	**ELA**	**SAL-EcTI**	**ELA-EcTI**
Neutrophils (cells/10^4^ μm^2^)	0.12 ± 0.05	0.51 ± 0.06 *	0.21 ± 0.07	0.22 ± 0.3 **
Macrophages (cells/10^4^ μm^2^)	0.30 ± 0.11	1.28 ± 0.17 *	0.74 ± 0.0.09	0.87 ± 0.12 **
TNF-α (cells/10^4^ μm^2^)	0.98 ± 0.19	3.92 ± 0.43 *	1.13 ± 0.23	2.11 ± 0.31 **
**Remodeling Markers**	**SAL**	**ELA**	**SAL-EcTI**	**ELA-EcTI**
Collagen Fibers (%)	9.69 ± 0.07	11.62 ± 0.37 *	9.3 ± 0.9	9.06 ± 0.7 **
Elastic Fibers (%)	0.29 ± 0.03	0.47 ± 0.03 *	0.34 ± 0.02	0.41 ± 0.04
MMP-9 (cells/10^4^ μm^2^)	3.17 ± 0.5	12.48 ± 0.86 *	4.69 ± 0.47	8.7 ± 1.42 *^/^**
MMP-12 (cells/10^4^ μm^2^)	5.03 ± 0.63	16.29 ± 1.07 *	5.93 ± 0.69	9.49 ± 0.37 *^/^**
TIMP-1 (cells/10^4^ μm^2^)	3.4 ± 0.33	14.04 ± 0.75 *	4.34 ± 0.4	11.3 ± 1.08 *^/^**
**Oxidative Stress Markers**	**SAL**	**ELA**	**SAL-EcTI**	**ELA-EcTI**
iNOS (cells/10^4^ μm^2^)	0.7 ± 0.15	3.33 ± 0.37 *	1.08 ± 0.23	1.94 ± 0.29 **
eNOS (cells/10^4^ μm^2^)	0.63 ± 0.18	3.01 ± 0.45 *	0.58 ± 0.12	1.51 ± 0.26 **
8-*iso*-PGF2α (%)	5.93 ± 0.58	13.89 ± 1.07 *	7.16 ± 0.9	11.08 ± 0.64 *^/^**

The neutrophils, macrophages, tumor necrosis factor-α (TNF-α), matrix metalloproteinase-9 (MMP-9), matrix metalloproteinase-12 (MMP-12), tissue inhibitor matrix metalloproteinase -1 (TIMP-1), inducible nitric oxide synthase (iNOS) and endothelial nitric oxide synthase (eNOS) are expressed in positive cells/10^4^µm^2^. The collagen fiber, elastic fiber, and 8-isoprostane-prostaglandin 2α (8-*iso*-PGF2α) are expressed as percentages (%). * *p* < 0.05, compared with the SAL and SAL-EcTI groups; ** *p* < 0.05, compared with the ELA group.

**Table 3:** Absolute values of the morphometric analysis for inflammatory, remodeling and oxidative stress markers in the airway walls.

**Inflammatory Markers**	**SAL**	**ELA**	**SAL-EcTI**	**ELA-EcTI**
Neutrophils (cells/10^4^ μm^2^)	0.89 ± 0.27	4.02 ± 0.38 *	0.87 ± 0.25	2.35 ± 0.40 *^/^**
TNF-α (cells/10^4^ μm^2^)	0.84 ± 0.38	4.01 ± 0.54 *	1.16 ± 0.22	2.45 ± 0.45 **
**Remodeling Markers**	**SAL**	**ELA**	**SAL-EcTI**	**ELA-EcTI**
Collagen Fibers (%)	4.46 ± 0.51	8.84 ± 0.44 *	3.15 ± 0.29	6.11 ± 0.7 **
Elastic Fibers (%)	0.30 ± 0.06	2.47 ± 0.36 *	0.91 ± 0.17	1.53 ± 0.35 **
MMP-9 (cells/10^4^ μm^2^)	4.61 ± 0.78	13.85 ± 1.53 *	4.4 ± 0.52	9.85 ± 0.82 *^/^**
MMP-12 (cells/10^4^ μm^2^)	5.25 ± 0.49	15.94 ± 0.95 *	6.05 ± 0.45	9.59 ± 0.48 *^/^**
TIMP-1 (cells/10^4^ μm^2^)	3.49 ± 0.65	13.13 ± 0.98 *	3.64 ± 0.79	11.82 ± 0.87 *
**Oxidative Stress Markers**	**SAL**	**ELA**	**SAL-EcTI**	**ELA-EcTI**
iNOS (cells/10^4^ μm^2^)	0.84 ± 0.37	5.13 ± 038 *	1.34 ± 0.27	2.39 ± 0.39 **
eNOS (cells/10^4^ μm^2^)	0.86 ± 0.28	3.09 ± 0.43 *	1.13 ± 0.2	1.99 ± 0.37 **
8-*iso*-PGF2α (%)	6.75 ± 0.39	16.12 ± 1.17 *	6.67 ± 0.89	12.04 ± 1.14 *^/^**
**Mucus Production**	**SAL**	**ELA**	**SAL-EcTI**	**ELA-EcTI**
MUC-5AC (cells/10^4^ μm^2^)	1.31 ± 0.48	7.09 ± 0.61 *	1.19 ± 0.27	3.5 ± 0.6 *^/^**

The neutrophils, TNF-α, MMP-9, MMP-12, TIMP-1, iNOS, eNOS and Mucin-5AC (MUC-5AC) are expressed in positive cells/10^4^ µm^2^. The collagen fiber, elastic fiber, and 8-*iso*-PGF2α are expressed as percentages (%) * *p* < 0.05, compared with the SAL and SAL-EcTI groups; ** *p* < 0.05, compared with the ELA group.

## Abbreviations

EcTI	*Enterolobium contortisiliquum* trypsin inhibitor
MMP-9	Matrix metalloproteinase-9
MMP-12	Matrix metalloproteinase-12
TIMP-1	Tissue inhibitor of matrix metalloproteinase-1
iNOS	Inducible nitric oxide synthase
eNOS	Endothelial nitric oxide synthase
TNF-α	Tumor necrosis factor-α
8-*iso*-PGF2α	8-isoprostane-PGF2α
MUC-5AC	Mucin-5AC
*R*_aw_	Airway resistance greater
*G*_tis_	Lower airway resistance or tissue
*H*_tis_	Lung tissue elastance
